# Status of deep learning for EEG-based brain–computer interface applications

**DOI:** 10.3389/fncom.2022.1006763

**Published:** 2023-01-16

**Authors:** Khondoker Murad Hossain, Md. Ariful Islam, Shahera Hossain, Anton Nijholt, Md Atiqur Rahman Ahad

**Affiliations:** ^1^Department of Computer Science and Electrical Engineering, University of Maryland Baltimore County, Baltimore, MD, United States; ^2^Department of Robotics and Mechatronics Engineering, University of Dhaka, Dhaka, Bangladesh; ^3^Kyushu Institute of Technology, Kitakyushu, Japan; ^4^Human Media Interaction, University of Twente, Enschede, Netherlands; ^5^Department of Computer Science and Digital Technology, University of East London, London, United Kingdom

**Keywords:** deep learning, EEG, BCI, future challenge, convolutional neural network (CNN)

## Abstract

In the previous decade, breakthroughs in the central nervous system bioinformatics and computational innovation have prompted significant developments in brain–computer interface (BCI), elevating it to the forefront of applied science and research. BCI revitalization enables neurorehabilitation strategies for physically disabled patients (e.g., disabled patients and hemiplegia) and patients with brain injury (e.g., patients with stroke). Different methods have been developed for electroencephalogram (EEG)-based BCI applications. Due to the lack of a large set of EEG data, methods using matrix factorization and machine learning were the most popular. However, things have changed recently because a number of large, high-quality EEG datasets are now being made public and used in deep learning-based BCI applications. On the other hand, deep learning is demonstrating great prospects for solving complex relevant tasks such as motor imagery classification, epileptic seizure detection, and driver attention recognition using EEG data. Researchers are doing a lot of work on deep learning-based approaches in the BCI field right now. Moreover, there is a great demand for a study that emphasizes only deep learning models for EEG-based BCI applications. Therefore, we introduce this study to the recent proposed deep learning-based approaches in BCI using EEG data (from 2017 to 2022). The main differences, such as merits, drawbacks, and applications are introduced. Furthermore, we point out current challenges and the directions for future studies. We argue that this review study will help the EEG research community in their future research.

## 1. Introduction

BCI is a method that uses psychology, electronics, computers, neuroscience, signal processing, and pattern recognition to work together. It is used to generate various control signals or commands from recorded brain signals of neural responses in order to determine the intentions of the medically challenged subject to perform a motor action to restore a quality of life. In a nutshell, the BCI turns the neural responses of the human brain into control signals or commands that can be used to control things such as prosthetic limbs, walking, neurorehabilitation, and movement. It is also used to assist medically challenged people with severe motor disorders, as well as healthy people, in their daily activities.

A generic BCI system (Schalk et al., 2004; Hassanien and Azar, 2015) comprises: (i) electrodes to obtain electrophysiological scheme patterns from a human subject; (ii) signal acquisition devices to record the neural responses of the subject's brain scheme; (iii) feature extraction to generate the discriminative nature of brain signals to decrease the size of data needed to classify the neural scheme; (iv) a translation algorithm to generate operative control signals; (v) a control interface to convert into output device commands; and (vi) a feedback system to guide the subject to refine specific neural activity to ensure a better control mechanism.

**Figure 1:**
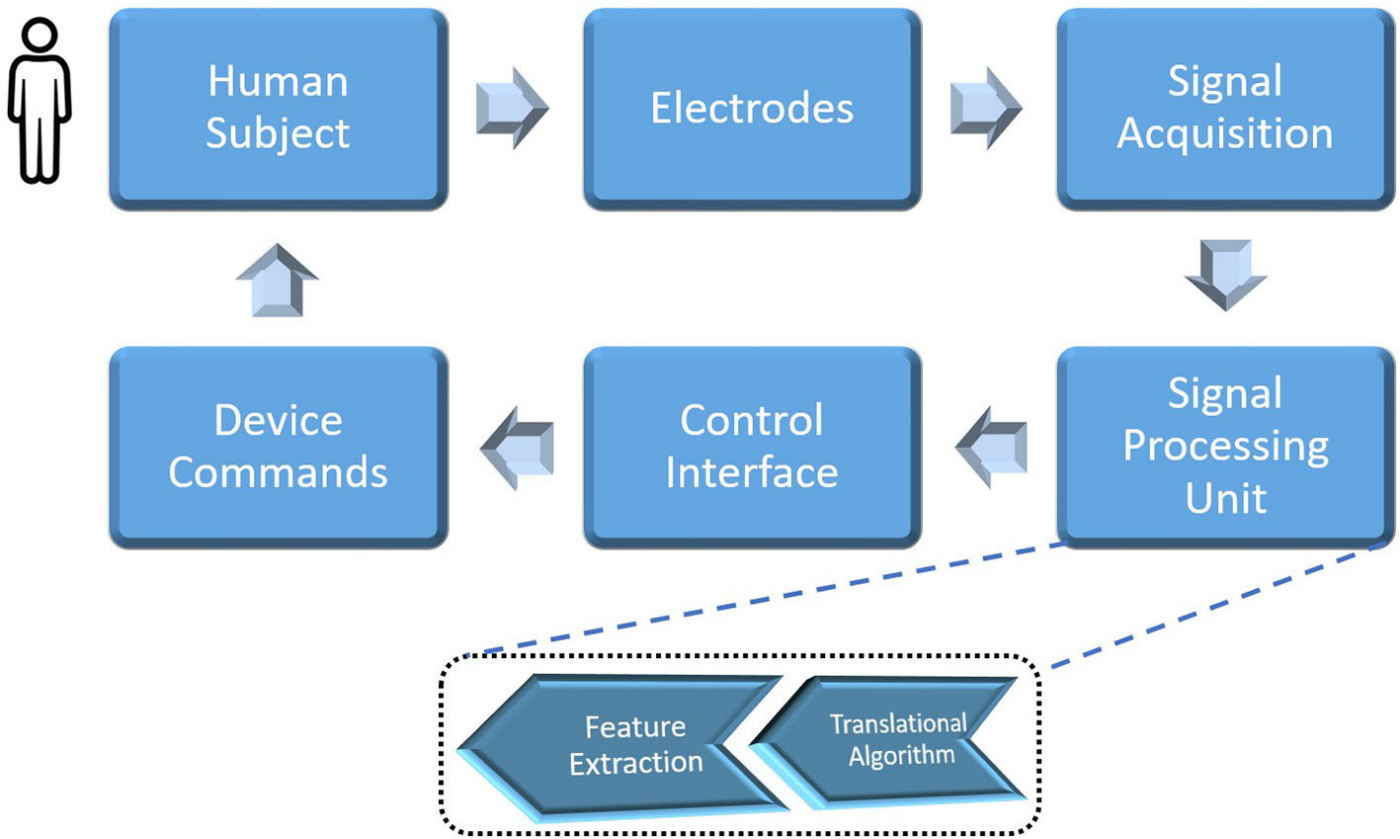
A generic brain–computer interface system.

On the other hand, there are two types of signal acquisition methods to trace neural activity, namely invasive and non-invasive methods (Schalk et al., 2004). A generic EEG-based BCI architecture is shown in Figure 1. Microelectrodes are neurosurgically implanted to the entire surface of the cerebral cortex or over the entire surface of the cerebrum under the scalp in an invasive method (Abdulkader et al., 2015). Even though this method gives high-resolution neural signals, it is not the best way to record neural activity from a human brain because it can cause scar tissue and infections.

In that case, the non-invasive method is preferred due to its flexibility and reduced risk. There are many techniques (Lotte et al., 2018) by which the neural activity is recorded, such as magnetoencephalography (MEG), functional magnetic resonance imaging (fMRI) (Acar et al., 2022; Hossain et al., 2022), and electroencephalography (EEG), and fully functioning near-infrared spectroscopy (fNIRS). The EEG method is preferred due to its robustness and user-friendly approach (Bi et al., 2013).

**Figure 2:**
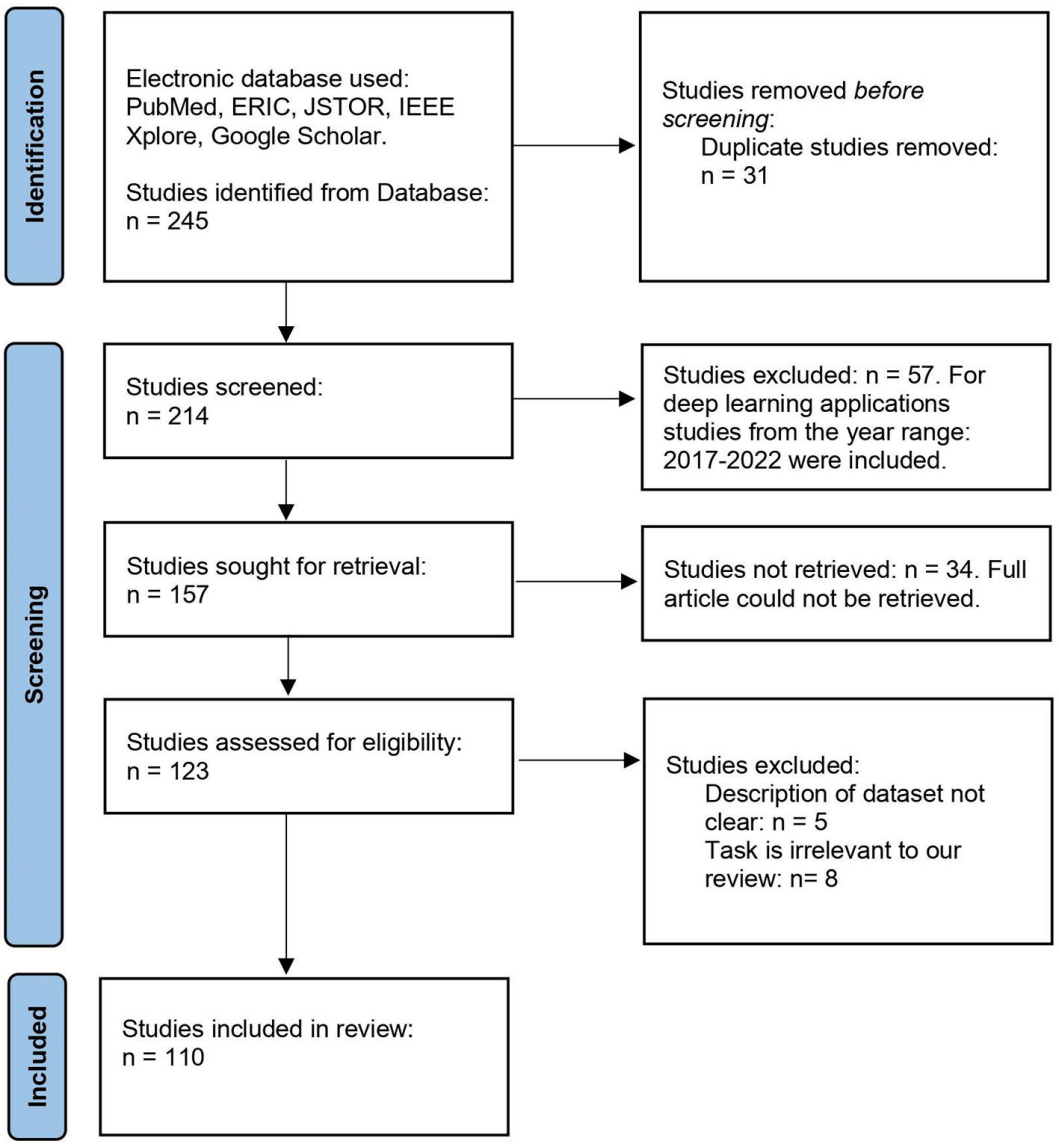
PRISMA flow diagram of the literature review process for studies on deep learning-based EEG-based BCI.

Artificial intelligence (AI) refers to systems or computers that imitate human intelligence to carry out tasks and can (iteratively) improve themselves depending on the information that they acquire. AI can take several forms, including machine learning and deep learning. Machine learning refers to the form of AI that can automatically adapt with only minimal intervention from humans. On the other hand, deep learning is a subset of machine learning that learns with large data by exploiting more neural network layers than classical machine learning schemes. There are several reviews on EEG-based BCI using signal processing and machine learning (Craik et al., 2019; Al-Saegh et al., 2021; Alzahab et al., 2021; Rahman et al., 2021; Wang and Wang, 2021). Nevertheless, machine learning reviews consist of a small part of deep learning modalities, so no review has focused exclusively on deep learning. One of the best things about deep learning is that it can do feature engineering on its own. In this method, the data are combed through by an algorithm that searches for features that correlate with one another, and then combines those features to facilitate faster learning without any explicit instructions. A comprehensive review is much anticipated as deep learning is the state-of-the-art classification pipeline. In this review, we report the most recent deep learning-based BCI research studies for the last 6 years. Figure 2 shows the PRISMA flow diagram of our literature review process.

We used PubMed, ERIC, JSTOR, IEEE Xplore, and Google Scholar as the electronic databases to get and retrieve the articles. As our goal is to include studies that relate to the three keywords: EEG data, BCI applications, and deep learning, we looked for studies that included all three keywords. From the 245 studies, we removed 31 as they were either fully duplicated or subversions of other articles. After screening the remaining 214 papers, we excluded 57 because they used deep learning only for related works or as only a part of the full pipeline, resulting in 157 studies. But we could not fully retrieve 34 studies out of 157, and this filtering gives us 123 articles, of which five do not have a clear dataset description, and the tasks of eight studies are irrelevant to our review. Finally, we explored 110 articles for this review.

To show how important this review is, we compare it to review that have been done recently in Table 1. As the comparison criteria, we have selected the coverage of the studies, the number of studies that are included in the review, the presence of dataset-specific studies in the review, whether the review is BCI application-specific, having future recommendations for the researchers, and whether the review is based only on deep learning. This study is the most recent study, which covers the articles until late 2022 and comprises the highest number of studies for the past 6 years. There has been no other review study that has done dataset-specific filtration of EEG-based BCI research, whereas we show the number of studies and results for each dataset separately. Furthermore, with a rich tabular comparison between the two works, we only consider the EEG data classification for BCI application-specific. Finally, we only concentrate on the deep learning algorithms for the EEG classification in contrast to most of the reviews.

**Table 1:** Comparison between previous review works and our proposed review study.

**References**	**Coverage**	**No. of studies**	**Dataset specific studies**	**Only BCI application?**	**Deep learning specific**	**Future recommendation**
Cao (2020)	2017–2020	Unspecified	No	Yes	No	No
Abiri et al. (2019)	1991–2017	Unspecified	No	Yes	No	No
Rahman et al. (2021)	2009–2021	54	No	No	No	Yes
Craik et al. (2019)	2014–2018	90	No	No	Yes	No
Alzahab et al. (2021)	2015–2020	47	No	Yes	Yes (hybrid deep learning)	No
Al-Saegh et al. (2021)	2016–2020	40	No	No	Yes	No
Wang and Wang (2021)	2016–2020	Unspecified	No	Yes	No	No
Our study	2017–2022	110	Yes	Yes	Yes	Yes

The study is organized as follows: After the introduction in Section 1, we introduce the core elements of EEG-based BCI in Section 2. Section 3 includes the classical methods, which have been exploited for EEG-based BCI tasks. Then, we analyzed the implementation of deep learning and related parts of this domain in Section 4. Sections 5, 6 are the discussion and conclusion of this article, respectively.

## 2. EEG-based BCI preliminaries

**Figure 3:**
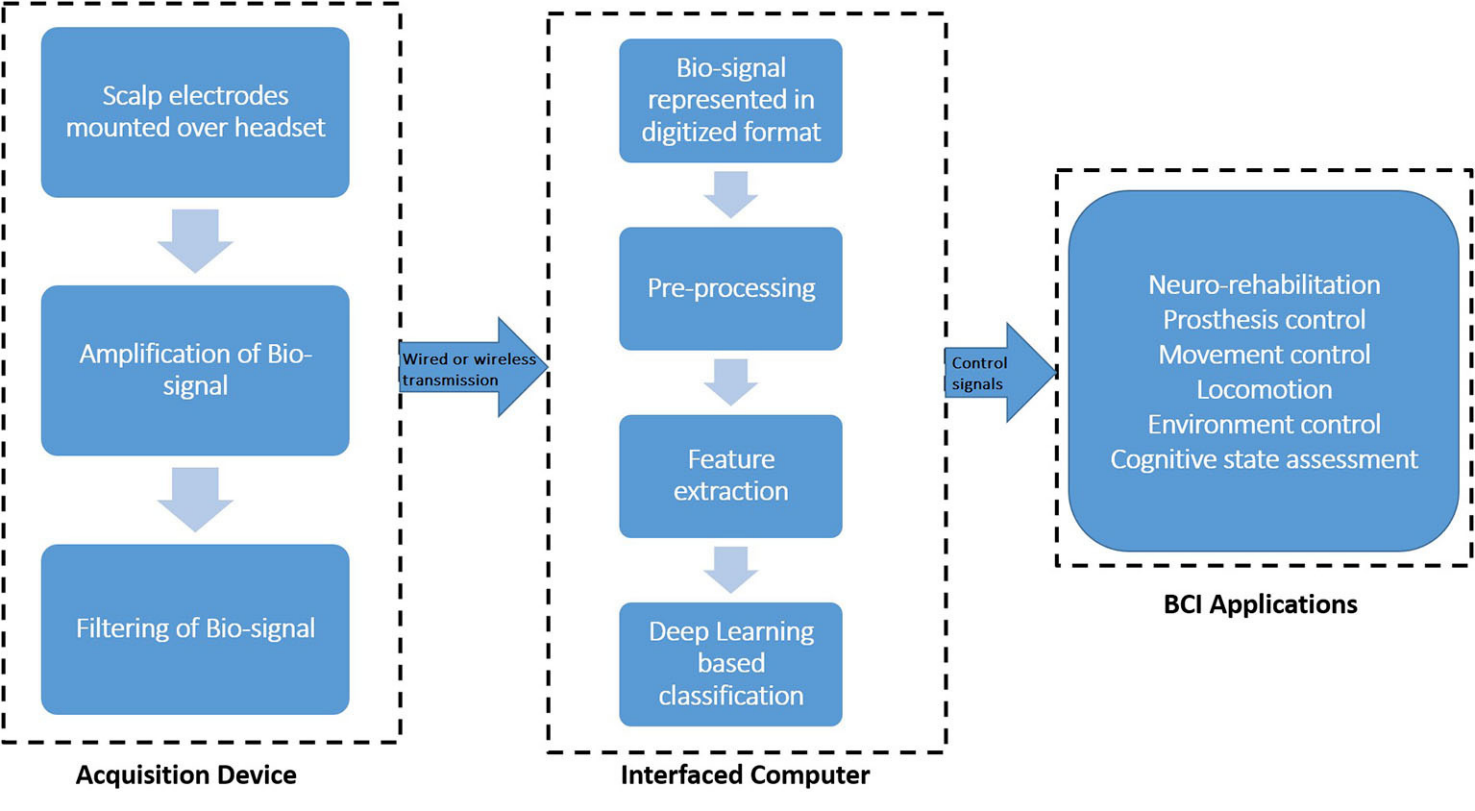
An architecture of BCI based on EEG data.

To translate mortal objectives or aspirations into real-time equipment control signals, the cognitive responses of humans are related to the physical world. In Figure 3, we depict the usage of EEG data in BCI applications. Electrophysiological activity patterns of human subjects are recorded by the acquisition device. Scalp electrodes are mounted over the headset to capture the neural responses of human subjects (Sakkalis, 2011). Furthermore, a pre-amplifier is used to make the brain signals stronger, and then the signal that has been strengthened is sent through a filter to get rid of unwanted parts, noise, or interference. After that, an analog-to-digital converter (ADC) converts the filtered analog signal to a digital signal. The electrical activities that had been recorded were then standardized to improve the signal-to-noise ratio (SNR) of the digital signal.

It is important to note that feature extraction gives you the things that neural activity cannot do. This means that you need less data to put the neural strategy into a category. Then, the data or information is put into a specific group or category of brain patterns. After this stage, the retrieved feature set is transformed into operational control signals. The control signals made in the previous step are used to control the external interface device. Thus, the BCI applications can be controlled by these command signals.

## 3. Classical methods for EEG-based BCI applications

EEG is by far the most prevalent strategy due to its high efficiency and usability (Schalk et al., 2004). Be that as it may, pattern-based control utilizing EEG signals is troublesome due to being exceedingly boisterous and containing numerous exceptions. The human neural impulses acquired from a BCI based on EEG include noise and other attributes in addition to the signal of neural activity. The challenges are getting rid of noise, trying to pull out relevant characteristics, and accurately classifying the signal. By translating the extracted feature set to give it a proper class label, operational commands can be made. There are two categories of classification algorithms: linear and non-linear classifiers (Guger et al., 2021).

The goal of quantitative classification is to figure out an object's system of classification based on how it looks. To recognize distinct types of brain activity, linear classifiers subscribe to the regime of trying to establish a linear relationship/function between both the dependent and independent variables of a classification method (Schalk et al., 2004). This set of classifiers involves linear discriminant analysis (LDA) and support vector machines (Wang et al., 2009). It sets up a hyperplane, which is a linear numerical operation that separates the different functions of the brain from the disentangled collection of characteristics.

Because of its simple, strong, and non-overfit operation and computing needs, the LDA, presuming the Gaussian distribution of data, has been implemented in several BCI platforms (Wang et al., 2009). Support Vector Machine (SVM) is a type of artificial intelligence that can be used for both regression and classification (Wang et al., 2009). Even though we mention regression issues, it is best suited for classification. The primary goal of the SVM algorithm is to track down a hyperplane in an N-dimensional space that evidently summarizes the data points. When no algorithmic solution can be found between the dependent and independent variables of the classification method, nonlinear classifiers are now used. Artificial neural networks (ANNs), k-nearest neighbor (KNN), and SVMs are some of these machine learning approaches (Lotte et al., 2018; Akhter et al., 2020; Islam et al., 2020).

The ANNs are broadly utilized in an assortment of classification and design acknowledgment assignments as they can memorize from preparing tests, and, in this way, classify the input tests in a like manner. These are the most broadly utilized ANNs for efficaciously characterizing multiclass neurological actions. They operate on the basis of conducting a preparatory calculation to adjust the weights pertaining to specific input and hidden layer neurons to minimize the violent square error (Wang et al., 2009).

Herman et al. (2008) conducted a classification of EEG-based BCI by investigating the type-2 fuzzy logic approach. They claimed that their model exhibited better classification accuracy than the type-1 model of fuzzy logic. They also compared this method with a well-known classifier based on LDA. On the other hand, Aznan and Yang (2013) applied the Kalman filter to an EEG-based BCI for recognizing motor visuals in an attempt to optimize the system's accuracy and consistency.

The quintessential dispersion (CSP) was used to collect the necessary information, and the radial basis function (RBF) was used to categorize the signal. They also compared their results with the LDA method and claimed that their RBF method showed a better result.

Zhang H. et al. (2013) linked Bayes classification error to spatial filtering, which is an important tool to extract and classify the EEG signal. They claimed that by validating the positive relationship between the Bayes error and the Rayleigh quotient, a spatial filter with a lower Rayleigh quotient measuring the ratio of power features could reduce the Bayes error. Zhang R. et al. (2013) proposed z-score LDA, an updated version of LDA that introduces a new decision boundary capable of effectively handling heteroscedastic class distribution-related classification.

Agrawal and Bajaj (2020) proposed a brain state signal measuring method based on non-muscular channel EEG to record the brain activity acting as a source to facilitate communication between a patient and the outside environment. They used fast and short-term Fourier transforms to decompose the signals obtained from neural activity into smaller segments. They implemented the classification tasks using a support vector machine. Depending on the values of the evaluation grades, the overall accuracy of the system was found to be approximately 92%. Pan et al. (2016) suggested a framework for a sentiment state detection system based on EEG-based BCI technology. They categorized two emotional responses, including happiness and sadness, using SVM. According to their observations, roughly 74.17% precision was noticed for such two classes.

Bousseta et al. (2018) proposed a BCI system based on EEG to control a robot arm by decoding the disabled person's thoughts obtained from the brain. They combined the principal component analysis with the fast Fourier transform to perform the feature extraction and then fed it to the radial basis function-based support vector machine as a classifier. The outputs of this classifier were turned into commands that the robot arm followed.

Amarasinghe et al. (2014) proposed a method consisting of three steps based on self-organizing maps to recognize neural activities for unsupervised clustering. They identified two thought patterns, such as moving forward and resting. They also implemented the classification process based on feed-forward ANNs. They claimed that their mapping methods showed approximately 8% improvement over ANN-based classification.

Korovesis et al. (2019) established an electroencephalography BCI system that controls the movement of a mobile robot in response to the eye blinking of a human operator. They used the EEG signals of brain activity to find the right features and then fed those features into a well-trained neural network to guide the mobile robot. They achieved an accuracy of 92.1%. Sulaiman et al. (2011) extracted distinguishing features for human stress from EEG-based BCI neural activity. They combined the power spectrum ratio of EEG and spectral centroid techniques to enhance the accuracy (88.89%) of the k-nearest neighbor (kNN) classifier, detecting and classifying human stress in two states, such as close-eye and open-eye.

**Figure 4:**
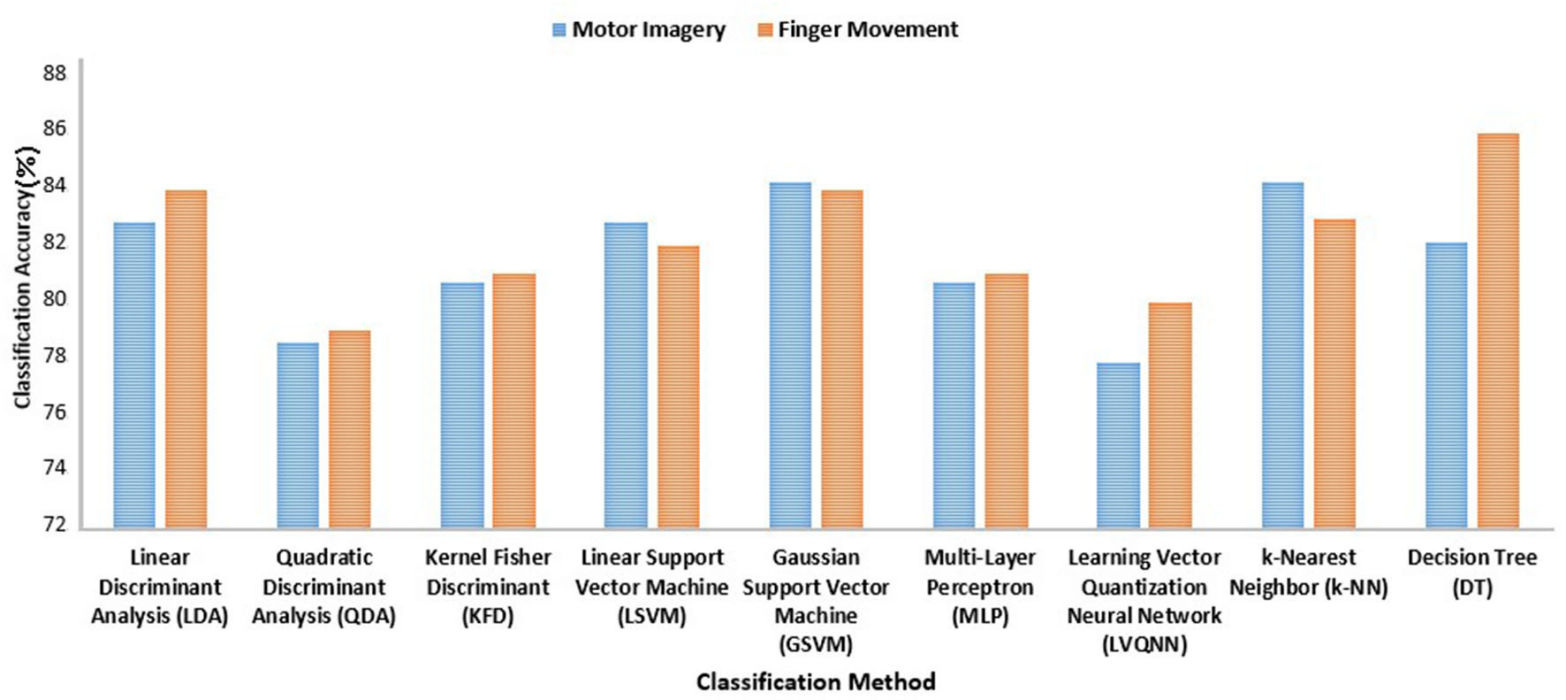
Classification algorithms and the corresponding accuracies of different classical classification methods based on a study.

Wang et al. (2009) conducted a review of various classification approaches for motor imagery (BCI competition III) and finger movement (BCI competition IV) on EEG signals. They compared the results in terms of the accuracy of the classification. Gaussian SVM (GSVM) and k-NN show the desired performance because these types of classification are more vigorous than nonlinear classifiers, as shown in Figure 4. However, learning vector quantization neural networks (LVQNN) and quadratic discriminant analysis (QDA) demonstrate the lowest accuracy. In addition, the performances of linear discriminant analysis (LDA) and linear SVM are almost identical. These demonstrate that the classical machine learning methods are not yet optimal for this domain. Therefore, we need to try out deep learning methods on large datasets in EEG-based BCI applications.

## 4. Utilizing deep learning in EEG-based BCI

Table 2 lists all the EEG-based BCI studies using deep learning for the last 6 years. We have listed the five most important parts of the studies: datasets, number of subjects, deep learning mode, BCI application, and classification result. This table will assist future researchers in determining the state of the art in this domain.

**Table 2:** EEG-based BCI studies using deep learning for the last 6 years.

**References**	**Dataset (No. of subjects)**	**Deep learning modality**	**Application**	**Classification result**
**EEG-based BCI with deep learning**				
Tang et al. (2017)	2 able-body subjects (2)	CNN	Classifying left hand and right hand movement	86.41%
Aznan et al. (2018)	4 subjects (4)	CNN	Classifying SSVEP frequencies	96.00%
Dose et al. (2018)	Physionet EEG MI Dataset (109)	CNN	Stroke rehabilitation	80.38%
El-Fiqi et al. (2018)	2 datasets (5 and 12)	CNN	Person identification	96.80%
Amber et al. (2019)	DRYAD (30)	CNN	Lie detection	99.60%
Nguyen and Chung (2018)	8 healthy subjects (8)	CNN	Developing a speller system	99.20%
Shoeibi et al. (2021)	21 patients with focal epilepsy (21)	CNN, LSTM	Diagnosing epileptic seizures	99.10% (CNN), 100% (LSTM)
Antoniades et al. (2018)	17 subjects (17)	CNN	Detecting epileptic discharges	68.00%
Völker et al. (2018)	Flanker task dataset (31)	CNN	Decoding error	81.70%
Behncke et al. (2018)	5 males and 6 females (11)	CNN	Decoding robot errors	75.00%
Oh et al. (2020)	20 Parkinson patients (20)	CNN	Identifying Parkinson Disease	88.25%
Zeng et al. (2018)	10 healthy subjects (10)	LSTM	Predicting mental states of drivers	91.79%
Hussein et al. (2019)	BCI (7)	LSTM	Detecting epileptic seizures	100%
Vilamala et al. (2017)	10 males and 10 females (20)	CNN	Scoring sleep stage	89–97%
Tabar and Halici (2016)	BCI competition IV dataset 2b (9)	CNN+SAE	Classifying right and left hand movement	72.40%
Olivas-Padilla and Chacon-Murguia (2019)	BCI competition IV dataset 2a (9)	CNN	Classifying MI	67.50% - 82.09%
Tayeb et al. (2019)	BCI competition IV dataset 2b (9)	CNN	Decoding MI movements	77.72%
Sundaresan et al. (2021)	8 with autism and 5 healthy subjects (13)	CNN+RNN	Classifying mental stress with autism	93.27%
Cai et al. (2021)	26 healthy subjects (26)	CNN	Classifying attentive state	72.73%
Ieracitano et al. (2021)	15 subjects (15)	CNN	Discriminating hand motion planning	76.21%
Reddy et al. (2021)	27 subjects (27)	CNN	Detecting drowsiness	85.42%
Petoku and Capi (2021)	462 trials of a single subject (1)	CNN	Detecting object movement	60.00%
Zhang et al. (2021)	BCI Competition IV dataset 2a and 2b (18)	CNN	Classifying MI	88.40%
Mai et al. (2021)	4 males and 2 females (6)	CNN	Detecting emotional states	93.34%
Deng et al. (2021)	BCI Competition IV 2a, III (12)	CNN	Classifying MI tasks	85.30%
Huang et al. (2022)	PhysioNet dataset (109)	CNN	Classifying MI	92.00%
Cho et al. (2021)	12 subjects (12)	Bi-LSTM	Classifying MI task	68.00%
Atilla and Alimardani (2021)	14 subjects while driving (14)	CNN	Classifying drivers attention	89.00%
Mammone et al. (2021)	15 participants (15)	CNN	Decoding motion planning	90.77%
Ak et al. (2022)	5 subjects (5)	CNN	Controlling robot manipulator	90.00%
Huang et al. (2021)	BCI competition IV dataset 2a (9)	CNN	Classifying MI	90.00%
Aldayel et al. (2020)	DEAP (32)	CNN	Classifying preference in neuromarketing	94.00%
León et al. (2020)	10 subjects (10)	CNN, RNN	Classifying SSMVEP signals	96.83%
Miao et al. (2020)	BCI competition IVa (5), right index finger MI dataset (10)	CNN	Classifying MI	90.00%
Ko et al. (2020)	SEED-VIG dataset (15)	CNN	Estimating driver vigilance	96.00%
Penchina et al. (2020)	11 subjects (11)	RNN, LSTM	Classifying anxiety in adolescents with autism	93.27%
Tortora et al. (2020)	11 healthy subjects walking on a treadmill (8)	LSTM	Decoding gait	AUC=90%
Rammy et al. (2020)	BCI Competition IV dataset 2a (9)	LSTM	Recognizing motor imagination	Mean kappa: 0.64
Liu J. et al. (2020)	DEAP (32), SEED (15)	CNN+SAE	Classifying emotion	92.86% (DEAP), 96.77% (SEED)
Li Y. et al. (2020)	EEGMMIDB (109)	Recurrent-CNN	Recognizing intention	97.36%
Maiorana (2020)	40 subjects (40)	RNN, CNN	Recognizing biometric	75.00%
Gao et al. (2020b)	DEAP (32), SEED (15)	CNN	Recognizing emotion	90.63%
Hwang et al. (2020)	SEED dataset (15)	CNN	Recognizing emotion	90.41%
Gao et al. (2020a)	15 right-handed healthy students (15)	CNN	Recognizing emotion	92.44%
Yang et al. (2020)	BCI competition IV dataset 1 (7)	CNN	Decoding MI EEG	86.40%
Fahimi et al. (2019)	120 healthy subjects performed the Stroop color test (120)	CNN	Detecting attention	79.26%
Tang et al. (2019)	BCI competition data IV 2a (9)	CNN+SAE	Classifying eMI task	79.90%
Roy et al. (2019)	BCI competition IV 2b dataset (9)	CNN	Classifying brain states	80.32%
Fares et al. (2019)	ImageNet-EEG (1)	Bi-LSTM	Classifying image	97.30%
Wilaiprasitporn et al. (2019)	DEAP dataset (32)	CNN, RNN	Identifying person	99.90%
Qiao and Bi (2019)	BCI competition IV 2a dataset (9)	CNN+Bi-GRU	Classifying MI	76.62%
Zgallai et al. (2019)	10 volunteers (10)	CNN	EEG-driven BCI smart wheelchair	70.00 (raw EEG), 96.00% (Fourier)
Gao et al. (2019)	8 subjects in fatigue states (8)	CNN	Evaluating driver fatigue	97.37%
Puengdang et al. (2019)	20 subjects (20)	LSTM	Authenticating person	91.44%
Song et al. (2019)	BCI Competition IV dataset 2a (9)	CNN	Classifying MI	73.40%
Zhao et al. (2019)	BCI Competition IV dataset 2a (9)	CNN	Classifying MI	Mean kappa: 0.64
Chen et al. (2019b)	DEAP dataset (32)	CNN	Recognizing emotion	AUC: 99.88%
Chen et al. (2019a)	157 subjects (157)	CNN	Identifying biometric	96.00%
Dai et al. (2019)	BCI Competition IV dataset 2b (9)	CNN+VAE	Classifying MI	Kappa = 0.60
Amin et al. (2019b)	BCI Competition IV dataset 2a (9)	CNN	Classifying MI	75.7%
Saha et al. (2019)	KARA (14)	CNN+LSTM	Categorizing phonology	77.90%
Ozdemir et al. (2019)	DEAP dataset (32)	CNN	Estimating emotional state	95.96%
Tiwari et al. (2021)	BCI competition V dataset (3), Emotiv dataset (16)	CNN	Classifying left hand and right hand task	72.51% (BCI V), 72.00% (Emotiv)
Dang et al. (2021)	CHB-MIT datasets (24)	CNN	Detecting epilepsy	99.56%
Polat and Özerdem (2020)	BCI competition 2003 (1)	CNN	Detecting cursor movements	90.38%
Chakladar et al. (2020)	STEW dataset (48)	Bi-LSTM	Estimating mental workload	82.57%
Li F. et al. (2020)	BCI Competition IV 2b (9)	CNN	Classifying MI	83.20%
Alazrai et al. (2019)	22 subjects (22)	CNN	Decoding MI tasks of the same hand	73.70%
Liu Y. et al. (2020)	DEAP dataset (32)	CNN	Recognizing emotion	95.27%
Arnau-González et al. (2017)	DREAMER dataset (23)	CNN	Identifying subject	94.01%
Zhu et al. (2022)	MBT-42 (42), Med-62 (62)	ConvNet, 3D-CNN	Classifying MI	73.12% (MBT-42), 72.66% (Med-62)
Mattioli et al. (2022)	EEG Motor Movement Dataset V 1.0.0 (109)	1D-CNN	Classifying MI	99.38%
Du and Liu (2022)	MRCP (12)	InceptionEEG-Net (CNN)	Classifying MI	AUC: 0.91%

It covers the key features, namely: datasets, number of subjects, deep learning modality, BCI application, and classification results.

### 4.1. Data preprocessing

Due to the presence of artifacts and contamination, EEG data arestill not being used for large-scale BCI studies (Pedroni et al., 2019). Even though some deep learning studies for EEG-based BCI say they did not use any preprocessing steps, most of the time, preprocessing steps are very important. Some research works combine the preprocessing steps in their deep learning pipeline and call it as end-to-end framework (Antoniades et al., 2018; Aznan et al., 2018; Zhang et al., 2021). Moreover, an additional CNN layer has been used for the preprocessing in some cases (Amin et al., 2019a; Tang et al., 2019).

Most of the time, frequency domain filters were used in research to limit the bandwidth of the EEG signal. This is useful when there is a specific frequency range of interest so that the rest can be safely ignored (Islam et al., 2016; Kilicarslan et al., 2016). In 30% of the studies, a signal below 45 Hz, or below a typical low gamma band, was low-pass filtered. The filtered frequency ranges were grouped by task type and artifact reduction methods. It shows that most research used a technique to get rid of artifacts along with lowering the frequency ranges that were studied.

From our observation, 20% of the studies manually eliminated artifacts (Rammy et al., 2020; Atilla and Alimardani, 2021; Sundaresan et al., 2021). It is easy to see unexpected outliers visually, such as when data are missing or significant EEG artifacts are evident. But it might be hard to tell the difference between noisy channels that are always on and noisy channels that are only noisy sometimes. Furthermore, since the way the data are processed is very random, it is hard for other researchers to copy the methods. In addition to this, 10% of the studies did not routinely eliminate EEG artifacts. Independent component analysis (ICA) (Delorme et al., 2007) and discrete wavelet transformation (DWT) were the most common artifact removal methods that were utilized in the remaining two-thirds of the analyzed research (Kline et al., 2015).

The EEG electrodes also take up undesired electrical physiological signals from eye blinks and neck muscles (Crespo-Garcia et al., 2008; Amin et al., 2019b). Additionally, there are issues with motion artifacts brought on by cable motion and electrode displacement while the individual is moving (Arnau-González et al., 2017; Chen et al., 2019a; Gao et al., 2019). There have been a lot of studies performed on how to find and remove EEG artifacts (Nathan and Contreras-Vidal, 2016), but it is not the primary focus of our review work. In summary, one of the three methods (i.e., manual process, automated process, or no removal of artifact) is considered in each study to conduct the artifact removal procedure.

### 4.2. Datasets

**Figure 5:**
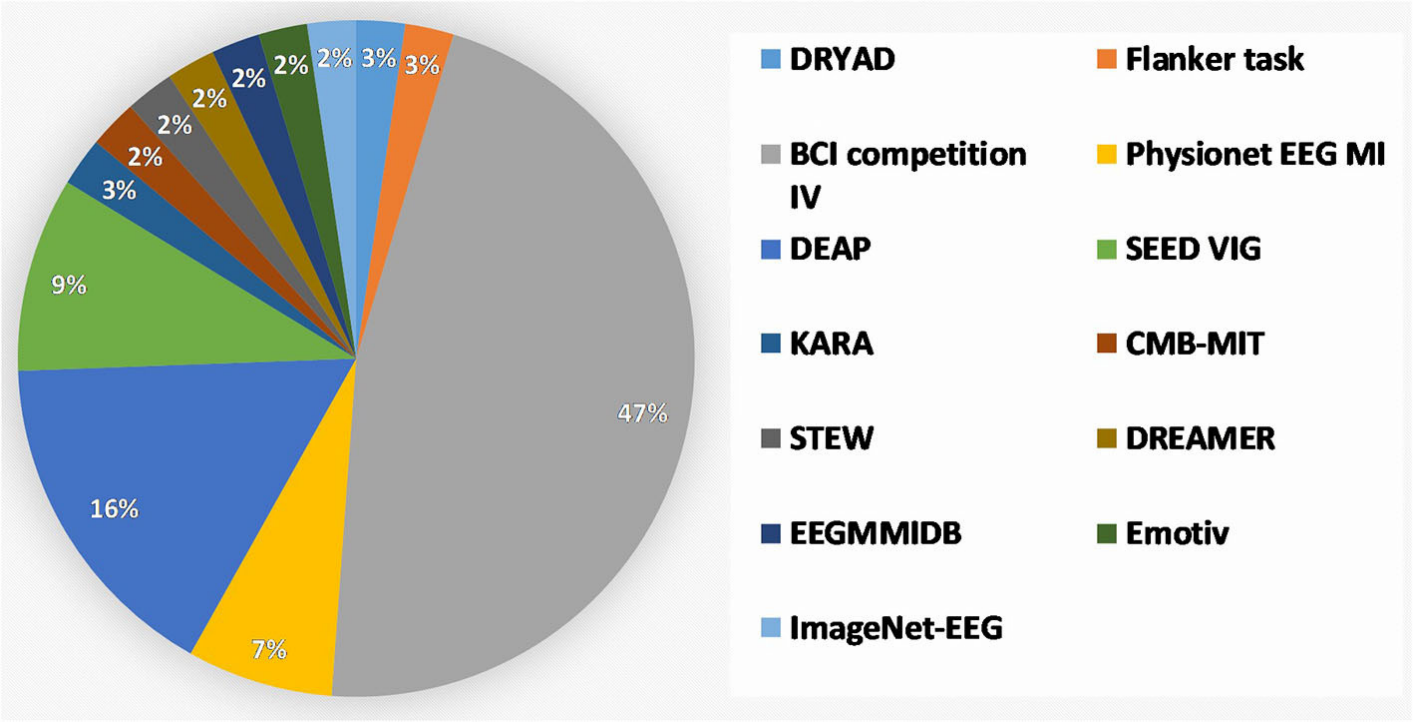
Distributions of datasets that are explored for EEG-based BCI applications.

One of the main limitations of the classical EEG-based BCI is the number of subjects who participated in this study. Within the course of this review, EEG-based datasets were covered. This scope was taken into account as keywords to find the right research articles on the Google Scholar and Research Gate websites. For this literature review, more than 100 research studies were found on these two websites by using the above criteria. Among these, around 47% of research has been conducted based on the BCI competition dataset. Moreover, 9%, 16%, and 7% of the studies have been conducted on DRYAD, SEED-VIG, and EEG MI datasets, respectively (Figure 5).

Deep learning has enabled larger datasets and more rigorous experiments in BCI. “How much data is enough data?” remains a significant question when using DL with EEG data. We looked at numerous descriptive dimensions to investigate this question: the number of participants, the amount of EEG data collected, and the task of the datasets. There are few studies that make use of their own collected datasets (Tang et al., 2017; Vilamala et al., 2017; Antoniades et al., 2018; Aznan et al., 2018; Behncke et al., 2018; El-Fiqi et al., 2018; Nguyen and Chung, 2018; Alazrai et al., 2019; Chen et al., 2019b; Fahimi et al., 2019; Hussein et al., 2019; Zgallai et al., 2019; Gao et al., 2020b; León et al., 2020; Maiorana, 2020; Penchina et al., 2020; Tortora et al., 2020; Atilla and Alimardani, 2021; Cai et al., 2021; Cho et al., 2021; Mai et al., 2021; Mammone et al., 2021; Petoku and Capi, 2021; Reddy et al., 2021; Shoeibi et al., 2021; Sundaresan et al., 2021; Ak et al., 2022). However, most of the deep learning studies have been conducted based on publicly available EEG datasets, such as:

- The dataset used to validate the classification method and signal processing for brain–computer interfaces was obtained from the BCI competition (Tabar and Halici, 2016; Amin et al., 2019b; Dai et al., 2019; Olivas-Padilla and Chacon-Murguia, 2019; Qiao and Bi, 2019; Roy et al., 2019; Song et al., 2019; Tang et al., 2019; Tayeb et al., 2019; Zhao et al., 2019; Li Y. et al., 2020; Miao et al., 2020; Polat and Özerdem, 2020; Rammy et al., 2020; Yang et al., 2020; Deng et al., 2021; Huang et al., 2021, 2022; Tiwari et al., 2021; Zhang et al., 2021). This dataset comprises EEG data obtained from participants. Class 1 was the left hand, Class 2 was the dominant hand, Class 3 was both feet, and Class 4 was the tongue in the cue-based BCI structure. For each subject, two workouts were captured on interspersing time frames. Each session consisted of six runs separated by relatively short pauses. A phase includes 288 efforts, with each effort being implemented 48 times.
- DRYAD dataset contains five studies that investigate natural speech understanding using a diversity of activities along with acoustic, cinematic, and envisioned verbal sensations (Amber et al., 2019).
- CHB-MIT dataset contains EEG recordings from children who have intractable seizures (Dang et al., 2021). After people stopped taking their seizure medicine, they were watched for up to a few days to find out more about their seizures and see if they were good candidates for surgery. There are 23 patients in the dataset, separated into 24 cases (a patient has 2 recordings, 1.5 years apart). There are 969 h of scalp EEG recordings in this dataset, comprising 173 seizures. Seizures of various sorts can be found in the dataset (clonic, atonic, and tonic).
- DEAP dataset (Koelstra et al., 2011) includes 32 individuals who saw 1-min long music video snippets and judged arousal/valence/like–dislike/dominance/familiarity, as well as the frontal facial recording of 22 out of 32 subjects (Chen et al., 2019b; Ozdemir et al., 2019; Wilaiprasitporn et al., 2019; Aldayel et al., 2020; Gao et al., 2020a; Liu J. et al., 2020).
- The SEED-VIG dataset integrates EEG data with diligence indicators throughout a driving virtual environment. In addition, there are 18 conductive gels and eye-tracking (Ko et al., 2020).
- SEED dataset wherein EEG was documented over 62 streams from 15 participants as they regarded short videos eliciting positive, negative, or neutral feelings (Gao et al., 2020a; Hwang et al., 2020; Liu J. et al., 2020).
- The STEW dataset includes the raw EEG data of 48 participants who took part in a multi-threaded workflow test using the SIMKAP experiment (Chakladar et al., 2020).
- One participant observes an arbitrary picture (chosen from 14k pictures in the ImageNet ILSVRC2013 training dataset) for 3 s, while their EEG signals are documented. Over 70,000 specimens are also included (Fares et al., 2019).

### 4.3. Deep learning modality

Deep Neural Networks (DNNs) are highly structured and therefore can learn features from unrefined or modestly heavily processed data, minimizing the need for domain-specific processing and feature extraction processes. Furthermore, DNN-learned attributes may be even more proficient or evocative than human-designed attributes. Second, as in many realms where DL has surpassed the previous condition, it has the potential to improve the effectiveness of other analyses and classifications. Third, DL makes it easier to make tasks such as conceptual sculpting and domain acclimation, which are not tried as often and fail less often when using EEG data. Deep learning techniques have made it feasible to synthesize high-dimensional structured data, such as images and audio.

Deep learning-based methods have been used to sum up high-dimensional, well-organized content such as images and speech. Computational methods could be used by readers to grasp transitional depictions or complement data. Deep neural networks combined with techniques such as linkage synchronization make it easier to learn representations that do not depend on the domain, while keeping information about the task for domain adaptation. Similar methods can be implemented with EEG data to obtain more accurate depictions, and as a result, improve the performance of EEG-based models across a wide range of subjects and tasks.

Various deep learning algorithms have been employed in EEG-based BCI applications, whereas CNN is clearly the most frequent one. For example, Arnau-González et al. (2017), Tang et al. (2017), Vilamala et al. (2017), Antoniades et al. (2018), Aznan et al. (2018), Behncke et al. (2018), Dose et al. (2018), El-Fiqi et al. (2018), Nguyen and Chung (2018), Völker et al. (2018), Alazrai et al. (2019), Amber et al. (2019), Amin et al. (2019b), Chen et al. (2019a,b), Fahimi et al. (2019), Gao et al. (2019), Olivas-Padilla and Chacon-Murguia (2019), Ozdemir et al. (2019), Roy et al. (2019), Song et al. (2019), Tayeb et al. (2019), Zgallai et al. (2019), Zhao et al. (2019), Aldayel et al. (2020), Gao et al. (2020a,b), Hwang et al. (2020), Ko et al. (2020), Li Y. et al. (2020), Liu J. et al. (2020), Miao et al. (2020), Oh et al. (2020), Polat and Özerdem (2020), Atilla and Alimardani (2021), Cai et al. (2021), Dang et al. (2021), Deng et al. (2021), Huang et al. (2021), Ieracitano et al. (2021), Mai et al. (2021), Mammone et al. (2021), Petoku and Capi (2021), Reddy et al. (2021), Tiwari et al. (2021), Zhang et al. (2021), Ak et al. (2022), and, Huang et al. (2022) have explored deep learning-based algorithms. However, more recent BCI studies have implemented other deep learning modalities including,

- Long short-term memory (LSTM) (Zeng et al., 2018; Fares et al., 2019; Hussein et al., 2019; Puengdang et al., 2019; Saha et al., 2019; Chakladar et al., 2020; Penchina et al., 2020; Rammy et al., 2020; Tortora et al., 2020; Cho et al., 2021; Shoeibi et al., 2021),
- Recurrent neural network (RNN) (Wilaiprasitporn et al., 2019; León et al., 2020; Li F. et al., 2020; Penchina et al., 2020; Mai et al., 2021; Sundaresan et al., 2021), and
- Autoencoders (AE) and variational AE (VAE) (Tabar and Halici, 2016; Dai et al., 2019; Tang et al., 2019).

## 5. Results and discussion

### 5.1. Dataset-specific studies

**Figure 6:**
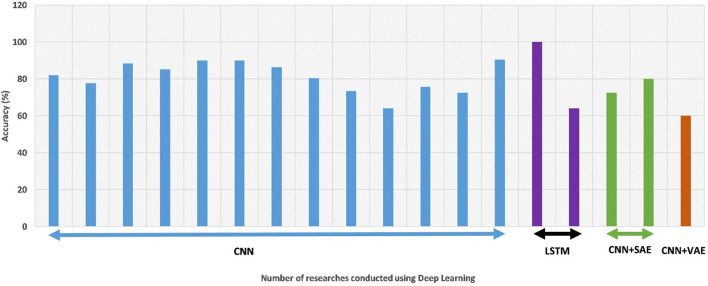
A comparative schematic of accuracies by various deep learning approaches [i.e., Convolutional neural network (CNN) (Islam et al., 2021), long short-term memory (LSTM), stacked autoencoder (SAE), and variational autoencoder (VAE)] on the BCI competition dataset.

Different classification algorithms give different maximum accuracy values for different datasets, as shown in Table 3. The LSTM algorithm gave the highest accuracy, which was based on the BCI competition dataset. All researchers achieved an accuracy of over 80% for this dataset, that is, this dataset has the highest accuracy so far. We have found the highest classification accuracy for any algorithm on the BCI competition dataset from various studies, as shown in Figure 6.

**Table 3:** Maximum accuracy obtained from different algorithms.

**References**	**Dataset**	**Max. accuracy (%)**	**Algorithms used**
Rammy et al. (2020)	BCI competition IV	100	LSTM
Wilaiprasitporn et al. (2019)	DEAP	99.90	CNN, RNN
Amber et al. (2019)	DRYAD	99.60	CNN
Dang et al. (2021)	CMB-MIT	99.56	CNN
Li Y. et al. (2020)	EEGMMIDB	97.36	R-CNN
Fares et al. (2019)	ImageNet-EEG	97.30	Bi-LSTM
Hwang et al. (2020)	SEED	96.77	CNN
Arnau-González et al. (2017)	DREAMER	94.01	CNN
Huang et al. (2022)	Physionet	92.00	CNN
Chakladar et al. (2020)	STEW	82.57	Bi-LSTM
Völker et al. (2018)	Flanker task	81.70	CNN
Saha et al. (2019)	KARA	77.90	CNN+LSTM
Tiwari et al. (2021)	Emotiv	72.00	CNN

**Figure 7:**
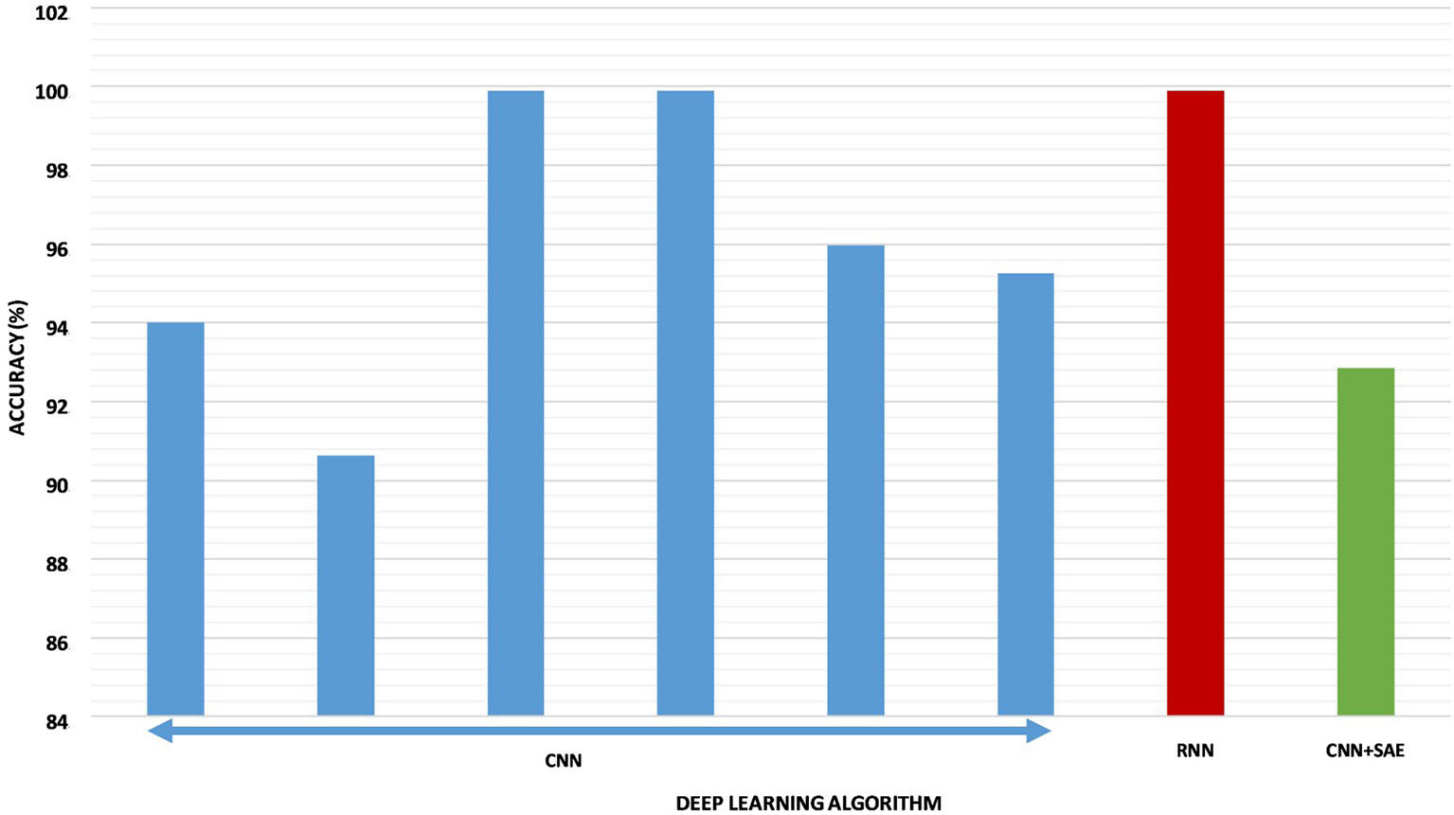
A graph of accuracies by various deep learning approaches on the DEAP dataset.

For the DEAP dataset (Koelstra et al., 2011), all researchers achieved an accuracy of roughly over 90% (Figure 7), that is, this dataset has the highest reliability so far. Unlike the previous dataset, this one has received little attention in terms of deep learning applications. As with the previous two datasets, there are a few works on the SEED dataset. However, the published works have achieved over 90% accuracy based on CNN or CNN+SAE (Gao et al., 2020a; Hwang et al., 2020; Liu J. et al., 2020). We can apply smarter algorithms to this dataset to explore further.

**Figure 8:**
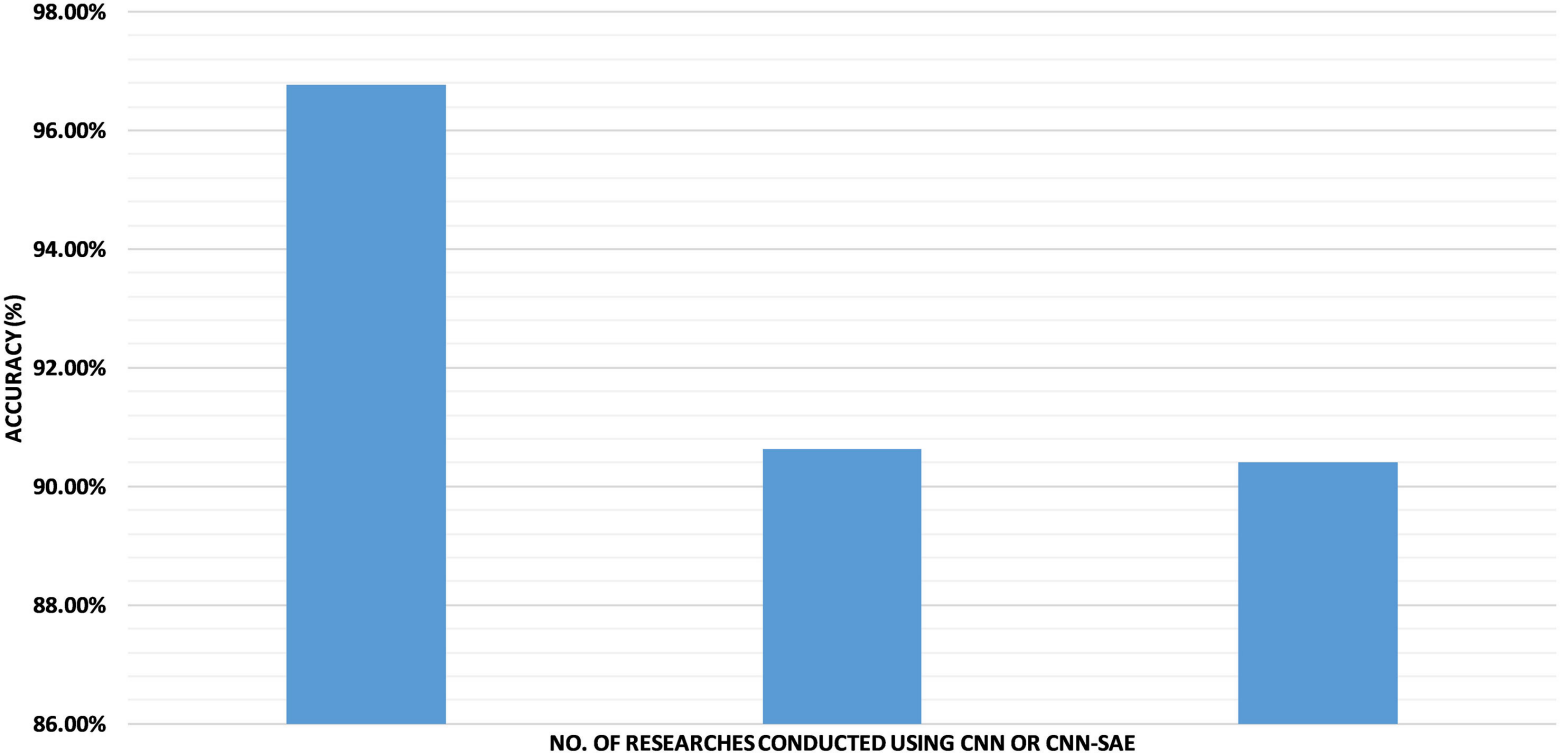
The accuracies of the SEED dataset based on CNN or CNN+SAE.

Due to insufficient work on the rest of the datasets shown in Figure 8, we cannot comment on them. However, we think that whether the accuracy can be increased on the rest of the dataset, it can be worked on in future.

### 5.2. Deep leaning models for BCI studies

Among the 110 publications that have been studied in this study, discriminative models, particularly CNN, are utilized most frequently. This is right since almost all BCI problems can be put into the category of classification problems. More than 75% of the models are powered by CNN algorithms, and we can summarize them as follows: (i) CNN can use EEG data to find hidden features and spatial correlations that can be used to classify something. As a result, CNN structures are used for classification in certain research while features are engineered in others; (ii) CNN has had considerable success in various research areas (especially in imaging and computer vision domains), making it exceedingly well-known and simple to use (through the available public code). Surprisingly, several BCI techniques naturally produce two-dimensional visuals that can be processed by CNN, and EEG data could be converted into two-dimensional images in the meantime for additional processing by CNN.

On the contrary, only 15% of the model-based articles used a recurrent neural network (RNN), even though RNN is capable of predicting temporal feature learning. One likely reason for this is that it takes time for an RNN to process a long sequence, and EEG signals are often long sequences. EEG signals, for example, are typically divided into 30-s segments with 2,500 time points at a 120 Hz sampling rate. Moreover, RNN takes more than 25 times as long to train as CNN for a sequence of 2,500 items.

Furthermore, among the typical models, the deep belief network (DBN), particularly the DBN-restricted Boltzmann machine (RBM), is the most often used model for feature extraction. DBN is commonly utilized in BCI for two reasons: 1) It is an efficient way to learn the top-down generative parameters that show how variables in one layer depend on variables in the layer above. 2) The values of the latent variables in each layer can be guessed by a single bottom-up pass that starts with an observed data vector in the bottom layer and uses the generative weights in the opposite direction. But most of the work that used the DBN-RBM model was published before 2018, which shows that DBN is not popular right now. Before 2018, researchers used DBN to learn about features, and then a classifier that did not use deep learning. Now, more and more studies use CNN or hybrid models for both learning about features and classifying them.

Finally, there are nine articles suggesting hybrid models for BCI research. Combinations of RNN and CNN account for approximately a third. It is logical to integrate RNN and CNN for both temporal and spatial feature learning, given that RNN and CNN are renowned for their exceptional temporal and spatial feature extraction capabilities. Combining representative and discriminative models is yet another sort of hybrid model. This is easy to understand since the first is used to pull out features and the second is used to put things into groups. There are nine articles that use this form of hybrid deep learning model, which encompasses almost all types of BCI signals. In addition, 12 studies have presented alternative hybrid models, including two discriminative ones. Several research, for instance, have advocated the combination of CNN with MLP in which the CNN structure is utilized to extract spatial data that are then given to an MLP for classification.

### 5.3. BCI applications and deep learning

Deep learning-based BCI systems are mostly used in the healthcare industry to identify and diagnose mental illnesses, including epilepsy, Alzheimer's disease, and other disorders (Dose et al., 2018). First, research focusing on sleep-stage recognition based on sleeping spontaneous EEG is utilized to identify sleeping disorders (Vallabhaneni et al., 2021). As a result, the researchers do not need to seek out patients with sleeping issues since it is simple to gather the sleeping EEG signals from healthy people in this condition. The diagnosis of epileptic seizures has also garnered a great deal of interest. The majority of seizure detection is dependent on spontaneous EEG and mental illness signs (Antoniades et al., 2018; Hussein et al., 2019; Dang et al., 2021; Shoeibi et al., 2021). CNN and RNN are common deep learning models in this context, as are hybrid models that combine RNN and CNN. Several methods (Turner et al., 2014) combined deep learning models for feature extraction with classical classifiers for detection. To diagnose seizures, researchers used a VAE in feature engineering followed by SVM.

Smart environments are a possible future application scenario for BCI. With the rise of the Internet of Things (IoT), BCI can be linked to a growing number of smart settings. For instance, an aiding robot may be used in a smart house (Zhang et al., 2018c) in which the robot is controlled by brain impulses. In addition, Behncke et al. (2018) examined how to operate a robot using visually stimulated spontaneous EEG and fNIRS data. BCI-controlled exoskeletons might assist individuals with compromised lower limb motor control in walking and everyday activities (Kwak et al., 2017). In future, research on brain-controlled equipment may be useful for developing smart homes and smart hospitals for the elderly and the crippled.

In comparison to other human–machine interface approaches, the greatest benefit of BCI is that it allows patients who have lost most motor functions, such as speaking, to interact with the outside world (Nguyen and Chung, 2018). Deep learning technology has considerably enhanced the efficiency of the brain's signal-based communications. The P300 speller is a common paradigm that allows individuals to type without a motor system, which can turn the user's intent into text (Cecotti and Graser, 2010). In addition, Zhang et al. (2018b) suggested a hybrid model that combines RNN, CNN, and AE to extract relevant characteristics from MI EEG to detect the letter the user intends to write. The suggested interface consists of 27 characters (26 English alphabets and the space bar) split into three character blocks (each block containing nine characters) in the first interface. There are three possible choices, and each one leads to a separate sub-interface with nine characters.

A prominent topic of interest for BCI researchers is the security industry. A security issue may be broken down into authentication (also known as “verification”) and identity (also known as “recognition”) components (Arnau-González et al., 2017; El-Fiqi et al., 2018; Chen et al., 2019b; Puengdang et al., 2019; Maiorana, 2020). The goal of the former, which is often a multi-class classification task, is to identify the test subject (Zhang et al., 2017). This is usually a simple yes-or-no question that only looks at whether the test subject is allowed or not. Existing biometric identification/authentication systems rely primarily on the unique inherent physiological characteristics of people (e.g., face, iris, retina, voice, and fingerprint). Anti-surveillance prosthetic masks that may defy face recognition, contact lenses that can fool iris detection, vocoders that can compromise speech identification, and fingerprint films that can fool fingerprint sensors are all vulnerable. Due to their great attack resilience, EEG-based biometric person identification systems are emerging as attractive alternatives. Individual EEG waves are almost impossible for an impostor to replicate, making this method extremely resistant to spoofing assaults faced by other identification methods. Deep neural networks were used by Mao et al. (2017) to identify the user's ID based on EEG signals, and CNN was used for personal identification. Zhang et al. (2017) presented and analyzed an attention-based LSTM model on both public and local datasets. The researchers (Zhang et al., 2018a) subsequently merged EEG signals with gait data to develop a dual-authentication system using a hybrid deep learning model.

Several articles simply aim to categorize the user's emotional state as a binary (positive/negative) or three-category (positive, neutral, and negative) issue using deep learning algorithms (Chen et al., 2019b; Ozdemir et al., 2019; Gao et al., 2020a,b; Hwang et al., 2020; Liu J. et al., 2020; Liu Y. et al., 2020; Sundaresan et al., 2021). Diverse articles employed CNN and its modifications to identify emotional EEG data (Li et al., 2016) and lie detection (Amber et al., 2019). Most of the time, the CNN-RNN deep learning model is used to find hidden traits in spontaneous emotional EEG. Using EEG data, Xu and Plataniotis (2016) employed a deep belief network (DBN) as a particular feature extractor for the emotional state categorization task. Moreover, on a more basic level, some studies seek to identify a positive/negative condition for each emotional dimension. For identifying emotions, Yin et al. (2017) suggested a multiple-fusion-layer-based ensemble classifier of AE. Each AE is made up of three hidden layers that remove unwanted noise from the physiological data and give accurate representations of the features.

For traffic safety to be assured, a driver must be able to keep up their best performance and pay close attention. It has been shown that EEG signals may be beneficial in assessing people's cognitive status while doing certain activities (Almogbel et al., 2018). A motorist is often considered alert if their response time is less than or equal to 0.7 s and weary if their reaction time is more than or equal to 2.1 s. By extracting the distinctive elements from the EEG data, Hajinoroozi et al. (2015) investigated the prediction of a driver's weariness. They investigated a DBN-based dimensionality reduction strategy. It is important to be able to tell when a driver is tired since that can make accidents more likely. Furthermore, it is practical to identify driver weariness in daily life. The technology that is used to record EEG data is easy to find and small enough to use in a car. In addition, the cost of an EEG headset is reasonable for the majority of individuals. Deep learning algorithms have greatly improved the accuracy of tiredness detection. In conclusion, driving sleepiness based on EEG may be identified with excellent accuracy (83–98%) (Fahimi et al., 2019; Ko et al., 2020; Atilla and Alimardani, 2021; Cai et al., 2021). The self-driving situation is where driver fatigue monitoring will likely be used in future. Since the human driver is often expected to react correctly to a request to intervene in most self-driving scenarios, the driver must always be aware. As a result, we think that using BCI-based drive fatigue detection can help the development of autonomous vehicles.

Human operators play an important role in automation systems for decision-making and strategy formulation. Human functional states, unlike those of machines or computers, cannot always meet the needs of a task because working memory is limited, and psycho-physiological experience changes over time. A lot of researchers have concentrated on this subject. The mental effort may be calculated using spontaneous EEG. Bashivan et al. (2015) introduced a DBN model, a statistical technique for predicting cognitive load from single trial EEG.

### 5.4. Recommendation for future research

However, there are still plenty of deep learning premises and domains to be used in EEG-based BCI, which will not only improve the performance but also make them more generalizable. Here are a few suggestions for future researchers regarding where they can uncover novelty utilizing deep learning.

- **Graph Convolutional Networks (GCNs)**: One of the fundamental functions of the BCI is controlling machines using only the MI and no physical motions. For the development of these BCI devices, it is very important to be able to classify MI brain activity in a reliable way. Even though previous research has shown promising results, there is still a need to improve classification accuracy to make BCI applications that are useful and cost-effective. One problem with making an EEG MI-based wheelchair is that it is still hard to make it flexible and resistant to differences between people. Traditional techniques to decipher EEG data do not include the topological link between electrodes. So, it is possible that the Euclidean structure of EEG electrodes does not give a good picture of how signals interact with each other. To solve the problem, graph convolutional neural networks (GCNs) are presented to decode EEG data. GCN is a semi-supervised model that is often used to get topological properties from data in non-Euclidean space. GCNs have been used successfully in a number of graph-based applications. Graphs can show complicated relationships between entities. GCN not only successfully extracts topological information from data but also it has interpretability and operability. Recently, researchers are shifting to GCN from CNNs for various applications as it can capture relational data better than CNNs. Though some studies have recently reported GCN in EEG-based BCI (Hou et al., 2020; Jia et al., 2020), it is mostly undiscovered. Any research in this domain using GCN might be the breakthrough needed to trigger deep learning-based BCI studies.
- **Transfer Learning**: The study of deep neural network-based methods for successfully transferring information from relevant disciplines is known as “deep transfer learning”. Transfer learning focuses on dealing with facts that defy this notion by utilizing knowledge acquired while completing one assignment for a different but related job. Transfer learning uses data that have already been used to increase the size of the dataset. This means that there is no need to calibrate from scratch, transferred information is less noisy, and TL can loosen BCI constraints. Session-to-session transfer learning in BCIs is based on the idea that features extracted by the training module and algorithms can be used to help a subject do the same task in a different session. To find the best way to divide decisions among the different training sections, it is important to look at what they all have in common. As TL has a lot more opportunities in BCI applications, we have a few recommendations for future researchers.
  The majority of TL research has focused on inter-subject and intersession transfer. Cross-device transfers are beginning to gain interest, although cross-task transfers are mostly unexplored. Since 2016, there has, to the best of our knowledge, been only one similar research (He and Wu, 2020). Transfers between devices and tasks would make EEG-based BCIs far more realistic.
  Utilizing the transferability of adversarial cases, adversarial assaults–one of the most recent advancements in EEG-based BCIs, may be carried out across several machine learning models. However, specifically considering TL across domains may boost the attack's performance further. In black box attacks, for example, TL can use publicly available datasets to reduce the number of queries to the victim model or better approximate the victim model with the same number of queries.
  Regression issues and emotional BCI are two fresh uses of EEG-based BCIs that have been piquing curiosity among researchers. It is interesting that they are both passive BCIs. Although affective BCI may be used to create both classification and regression problems, the majority of past research has been on classification issues.
- **Generative Deep Learning** : The primary purpose of generative deep learning models is to produce training samples or supplement data. In other words, generative deep learning models help the BCI industry by making the training data better and giving it more of it. After augmenting the data, discriminative models will be used for classification. This method is meant to make trained deep learning networks more reliable and effective, especially when there is not a lot of training data. In short, the generative models use the input data to make a set of output data that is similar to the input data. This section will present two common generative deep learning models: variational autoencoder (VAE) and generative adversarial networks (GANs).
  VAE is an important type of AE and one of the best algorithms for making things. The standard AE and its variations can be used for representation, but they cannot be used for generation since the learned code (or representation) might not be continuous. Therefore, it is impossible to make a random sample that is the same as the sample that was put in. In other words, interpolation is not supported by the standard AE. Therefore, we can duplicate the input sample but cannot construct one that is similar. This trait is what makes VAE so valuable for generative modeling: the latent spaces are meant to be continuous, which can make a huge contribution to capturing EEG data features for BCI applications (Lee et al., 2022).
  Machine learning and deep learning modules must be trained on a significant amount of real-world data to perform classification tasks; however, there may be restrictions on obtaining enough real data or the time and resources required may be simply too great. GANs, have seen an increase in activity in recent years, and are primarily used for data augmentation to address the issue of how to produce synthetic yet realistic-looking samples to mimic real-world data using generative models so that the training data sample number can be increased. In comparison to CNNs, GANs have, to the best of our knowledge, been studied much less in BCIs. This is primarily due to the incomplete evaluation of the viability of using a GAN to generate time sequence data. The spatial, spectral, and temporal properties of the EEG data produced by the GAN are comparable to those of actual EEG signals (Fahimi et al., 2020). This opens up new avenues for future research on GANs in EEG-based BCIs.

## 6. Conclusion

Deep learning (DL) has historically resulted in significant breakthroughs in supervised classification tasks, which were envisaged to be the concentration of the majority of research chosen for assessment. Remarkably, numerous studies spotlighted the new use cases facilitated by the study results. For example, generating visual effects based on EEG, deriving EEG, learning from other participants, and learning about attributes are all different ways to learn. One of the main reasons for using DL is that it can manage raw EEG data without mandating a substantial preprocessing step, which is alluded to in the literature as an “end-to-end structure.” Given that EEG is clearly linked to certain parts of the brain, we thought that RNNs would be much more widespread than models that do not explicitly take time into account.

Adding to its prospects is the willingness of deep learning in the EEG to extrapolate across respondents and facilitate transfer learning across activities and domains. Regardless of the fact that intra-subject models are still the most efficacious when only restricted evidence is accessible, ensemble learning may well be the best way to overcome this restriction given the obvious determining factor of the rate of EEG data. Using a predictive model, one can train a neural network on a sample of subjects before fine-tuning it on a single individual, which is likely to result in favorable results with less data from the individual. DNNs are typically regarded as “black boxes” when likened to more conventional means; therefore, it is crucial to scrutinize trained DL models. Indeed, simple model inspection techniques such as showing the weights of a linear classifier do not apply to deep neural networks, making their decisions far more difficult to comprehend.

This study presents an overview of EEG-based BCIs incorporating deep learning, with a concentration on the epistemological advantages and pitfalls, as well as the invaluable efforts in this area of study. This study shows that more research needs to be conducted on how much data are needed to use deep learning in EEG processing to its fullest potential. This type of research could look at the relationship between performance and data volume, effectiveness and data augmentation, performance, data volume, and network depth. For each BCI application, researchers have examined measurement techniques, control signals, EEG feature extraction, classification techniques, and performance evaluation metrics. Tuning hyper-parameters could have been the key to increasing the efficiency of deeper frameworks in deep learning mode by adjusting hyper-parameters. As mentioned earlier about the lack of hyper-parameter search in this domain, this issue should be addressed in future studies.

## Author contributions

KH, SH, and MI contributed the core writing and analysis. AN and MA edited and partially wrote the paper. All authors contributed to the article and approved the submitted version.
